# Paired DNA/RNA testing uncovers a deep intronic *PTEN* pathogenic variant associated with clinical Cowden Syndrome: a case report

**DOI:** 10.3389/fonc.2025.1679432

**Published:** 2025-10-09

**Authors:** Michael J Hall, Dong Won Kim, Devang Namjoshi, Michelle McSweeny, Marcy E Richardson, Ashley PL Marsh, Felicia Hernandez, Demitrios Dedousis

**Affiliations:** ^1^ Department of Clinical Genetics, Cancer Prevention and Control Program, Fox Chase Cancer Center, Philadelphia, PA, United States; ^2^ Internal Medicine, Saint Vincent Hospital, Worcester, MA, United States; ^3^ Ambry Genetics, Aliso Viejo, CA, United States

**Keywords:** case study, clinical PTEN Hamartoma Syndrome, clinical Cowden Syndrome, DNA/RNA testing, hereditary cancer syndrome

## Abstract

Identification of a deep intronic *PTEN* pathogenic variant, which was not detected by standard DNA-targeted panel sequencing but was uncovered by targeted *PTEN* RNA sequencing using CaptureSeq technology, illustrates the added value of concurrent DNA and RNA analyses in risk assessment for *PTEN* Hamartoma Tumor Syndrome (PHTS) and in patients given the diagnosis of clinical Cowden Syndrome (CS). These findings have significant clinical implications, including providing the rationale for testing patients meeting clinical criteria for PHTS/CS with concurrent DNA and RNA testing. It also supports reevaluation of patients who test negative by DNA testing alone but with a clinical diagnosis of PHTS/CS with subsequent RNA testing to identify and clinically interpret previously undetected deep intronic *PTEN* variants. Where cancer treatment and prevention decisions hinge on correct diagnoses, concurrent DNA and RNA testing rather than stepwise testing can permit faster, more accurate results and earlier clinical actionability.

## Introduction

DNA-based multigene panel testing is a commonly used approach to detect germline pathogenic variants (PV) in hereditary cancer risk genes in patients with or at risk for inherited cancers. However, non-diagnostic results are often reported on clinical testing despite personal or family history strongly suggestive of familial risk and/or meeting criteria for a clinical cancer syndrome. Identifying PVs associated with hereditary cancer risks has important management implications for patients, including decisions related to screening, surgical prophylaxis, and chemoprevention. These results also have important implications for close family members, who may be diagnosed with a hereditary cancer predisposition prior to developing a cancer, allowing a positive relative to use surveillance protocols, and chemoprevention to reduce their risk of cancer. Moreover, a relative may find that a familial risk was not inherited, freeing them from the need to conduct close cancer surveillance.

There has been significant interest in RNA-based genetic testing to clarify clinical actionability of hereditary cancer gene variants that potentially impact RNA splicing but are classified as inconclusive by DNA testing alone. Splicing refers to the process of removing non-coding sequences (introns) from an RNA molecule followed by the ligation of the adjoining exons. However, DNA variants, including those situated deep within an intron, can result in mis-splicing (1). DNA testing in its current form is limited, as it typically captures only exons and short stretches (5–20 base pairs) of the flanking introns. Early reports have suggested that the addition of RNA testing to DNA only sequencing among patients with non-diagnostic testing may significantly increase testing yield. A recent study reported a 9.1% increase in the detection of PV with complementary DNA and RNA testing (1). Another important application of RNA testing is the ability to detect deep intronic variants. Compared with traditional DNA sequencing, RNA testing increases our ability to detect and interpret mis-splicing and alternate splicing due to deep intronic variants. It has been estimated that approximately 15% to 25% of DNA variants in genes associated with hereditary cancer disrupt RNA splicing but that these alterations are often classified as variants of uncertain significance (VUS) by clinical laboratories (2). One approach has been to utilize *in silico* models to predict splicing impact. However, in one study, *in silico* models incorrectly predicted splicing impact for 16%-25% of the 64 studied variants and failed to make a prediction for another 2%-9% of the variants (2). Conducting RNA testing as a supplement to DNA testing led to clarification of 88% (49 of 56 cases) of inconclusive results (2). These results suggest that *in silico* models may not be an adequate substitute for RNA testing and cannot be used alone to support clinical decision making.

*PTEN* hamartoma tumor syndrome (PHTS) is a family of related genetic disorders including Cowden syndrome (CS), Bannayan–Riley–Ruvalcaba Syndrome (BRRS), adult Lhermitte–Duclos disease, and autism spectrum disorders associated with macrocephaly (3). PHTS also includes Proteus-like syndrome, which refers to those individuals with some clinical features of PTEN-related Proteus syndrome such as skeletal overgrowth but lack all required criteria for diagnosis. Germline *PTEN* alterations are not identified in all individuals with PHTS. Estimates vary, but germline *PTEN* alterations may remain unidentified in approximately 40% of individuals with a clinical diagnosis of BRRS (4), 80% of individuals with Proteus syndrome (4), and 15% of individuals with classic CS (4). While it is conceivable there are other non-*PTEN* genetic variants to explain these cases, there is also likely a percentage of patients with germline *PTEN* alterations not currently captured by DNA testing alone.

In the current report, we present the case of an adult female patient with clinical Cowden Syndrome who had negative targeted DNA sequencing but was found on concurrent RNA testing (i.e., targeted RNA sequencing with CaptureSeq technology) to have a likely pathogenic, deep intronic splice variant in *PTEN*. The goal of this clinical case presentation is to highlight the relevant clinical features of this case, including multiple cancers associated with clinical Cowden syndrome and the striking gastrointestinal polyposis phenotype, and to bring to attention the value of paired RNA testing among patients with a clinical CS or PHTS diagnosis who have had negative DNA-only testing or who remain untested.

## Results

### Patient characteristics

The patient is a 64-year-old woman of German/Scottish ancestry with a personal history of breast cancer diagnosed first at age 42 (right breast DCIS, treated with lumpectomy and radiation therapy) and then again at age 53 (left breast, ER/PR positive Stage IIA invasive ductal breast cancer, treated with bilateral mastectomy and TRAM flap and docetaxel and cyclophosphamide chemotherapy followed by extended hormone therapy) (**Table 1**). She also has a history of right thyroid gland lobectomy at age 54 due to multinodular goiter with microinvasive papillary cancer of the thyroid in 2012.

**Table 1:** Provides a timeline of diagnosis of PHTS features with additional clinically relevant details.

Patient details		
PHTS feature	Age of diagnosis	Details
Right breast cancer	42	DCIS, s/p lumpectomy and RT
Left breast cancer	53	Stage IIA IDC, s/p bilateral mastectomy, adjuvant chemotherapy, hormone therapy
Thyroid goiter & cancer	54	Microinvasive papillary carcinoma, s/p R thyroid lobectomy
Ganglioneuromatosis of colon	63	27 total polyps resected through entire colon; some hyperplastic and lymphoid aggregates also noted *Of note, colonoscopy done at age 58 noted extensive "inflammatory" polyposis of colon
Oral condyloma	~40's	s/p resection
Macrocephaly	n/a	Head circumference: 59.5 cm
Trichilemmomas, oral papules, acrochordons	n/a	Noted on exam; no biopsy done
Genetic testing details: CancerNext-Expanded panel (77 genes) with RNAinsight resulted 12/29/2022. Panel included paired DNA/RNA testing for 75 of these genes through Ambry Genetics.	Patient initially referred for genetic counseling/testing at age 64 in June 2022, a year after her colonic ganglioneuromatosis was noted. After record review, she was noted to have extensive "hyperplastic/inflammatory" polyposis at age 58 but was not referred for genetic evaluation at that time.	
Patient reaction to results: Felt positive *PTEN* result explained much of her unusual medical history. Enthusiastic about research; trained as RN and focused career on teaching nursing students. Endorsed sharing clinical photos for research purposes. Requested copy of poster presentation from CGA-IGC conference 2024 highlighting her case and any subsequent publications based on her case.		

**Figure 1:**
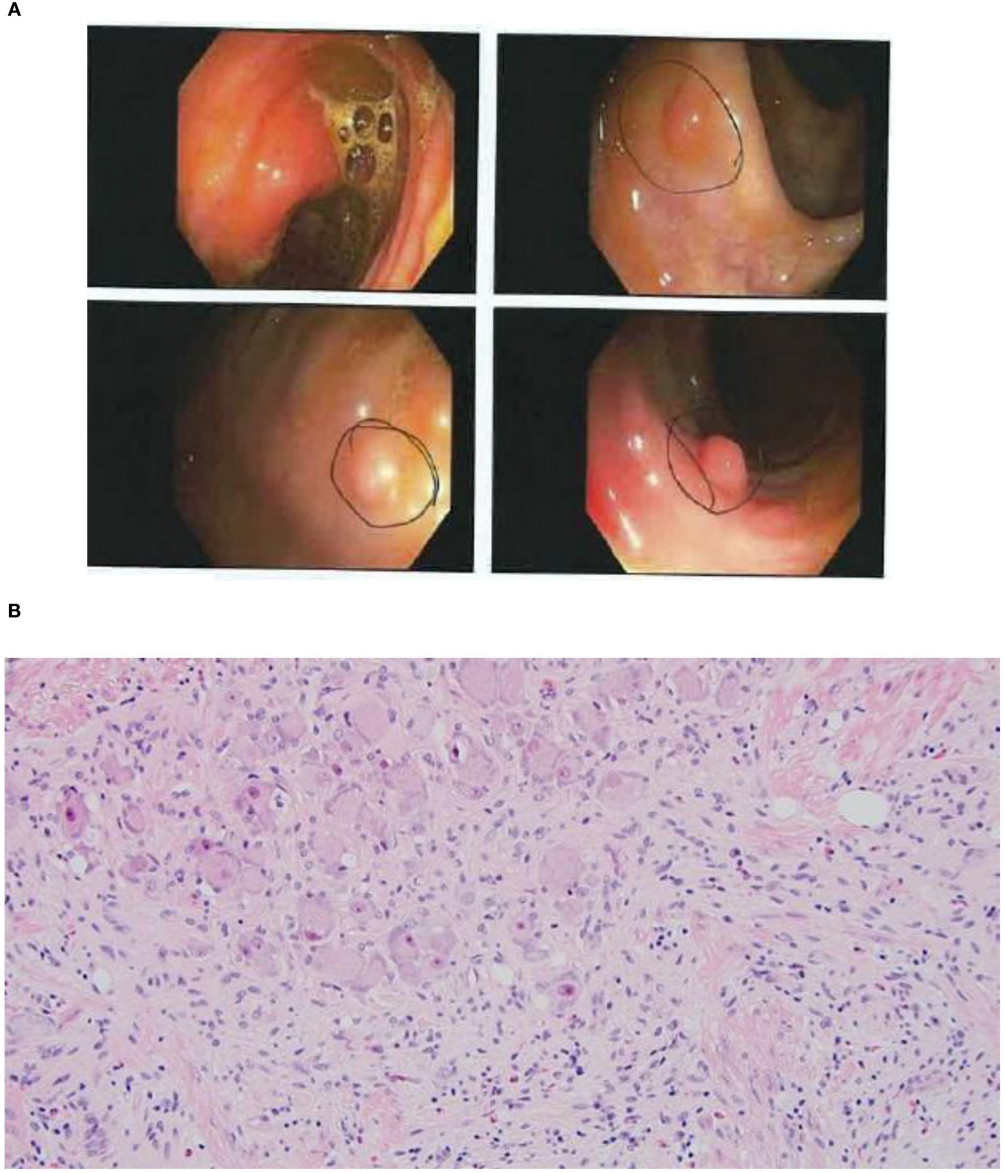
**(A)** Still images of the patient's colon captured on her second colonoscopy. Sub-centimeter ganglioneuromas were identified in multiple regions of the colon. **(B)** High-power microscopic view of ganglioneuromas.

Due to a history of chronic normocytic anemia (Hg 10.7-11.6, MCV 97-101), the patient was referred for colonoscopy in 2021. Colonoscopies performed in 2021 and 2022 identified diffuse colon polyposis with at least 16 and 11 polyps identified, respectively. These polyps were resected and classified by microscopic examination, with the majority classified as ganglioneuromas and several classified as lymphoid aggregates (see **Figures 1A, B**). Upper endoscopy performed in 2022 showed a negative result for gastric ulcers or evidence of *H. pylori* infection, with additional pertinent negative results of no upper gastrointestinal ganglioneuromas, hamartomas, or esophageal glycogenic acanthoses.

In 2022, the patient was referred to our center for genetic risk assessment and genetic testing due to her recently discovered polyposis with ganglioneuroma dominance. During her genetic counseling visit, a comprehensive history was taken to investigate colonic polyposis and PHTS-associated features. Family history included a father with colorectal cancer (age 62) and lung cancer (age 65), a sister with breast cancer (age 72), a brother with liver cancer (age 61), a paternal grandmother with neuroblastoma (age 52), and a paternal grandfather with lung cancer (age 68). The patient (G2P2) had two sons without a history of cancer, the younger of whom was reported to have had a history of multiple skin lipomas diagnosed in his 40s. There was no family history of autism or intellectual disability. The patient was not aware of any diagnosis of macular pigmentation of the glans penis in close male relatives.

The patient denied knowledge of trichilemmomas of the skin, palmoplantar keratoses, oral mucosal papillomatosis, or verrucous facial papules. She did, however, report a remote history of oral condyloma removed surgically several years earlier and had in the 1–2 years prior to genetic assessment seen a dermatologist to have multiple axillary skin tags removed. She denied knowledge of any intracranial and/or developmental venous anomalies. The patient reported a history of nephrolithiasis and prior history of hydronephrosis leading to unilateral atrophic kidney.

**Figure 2:**
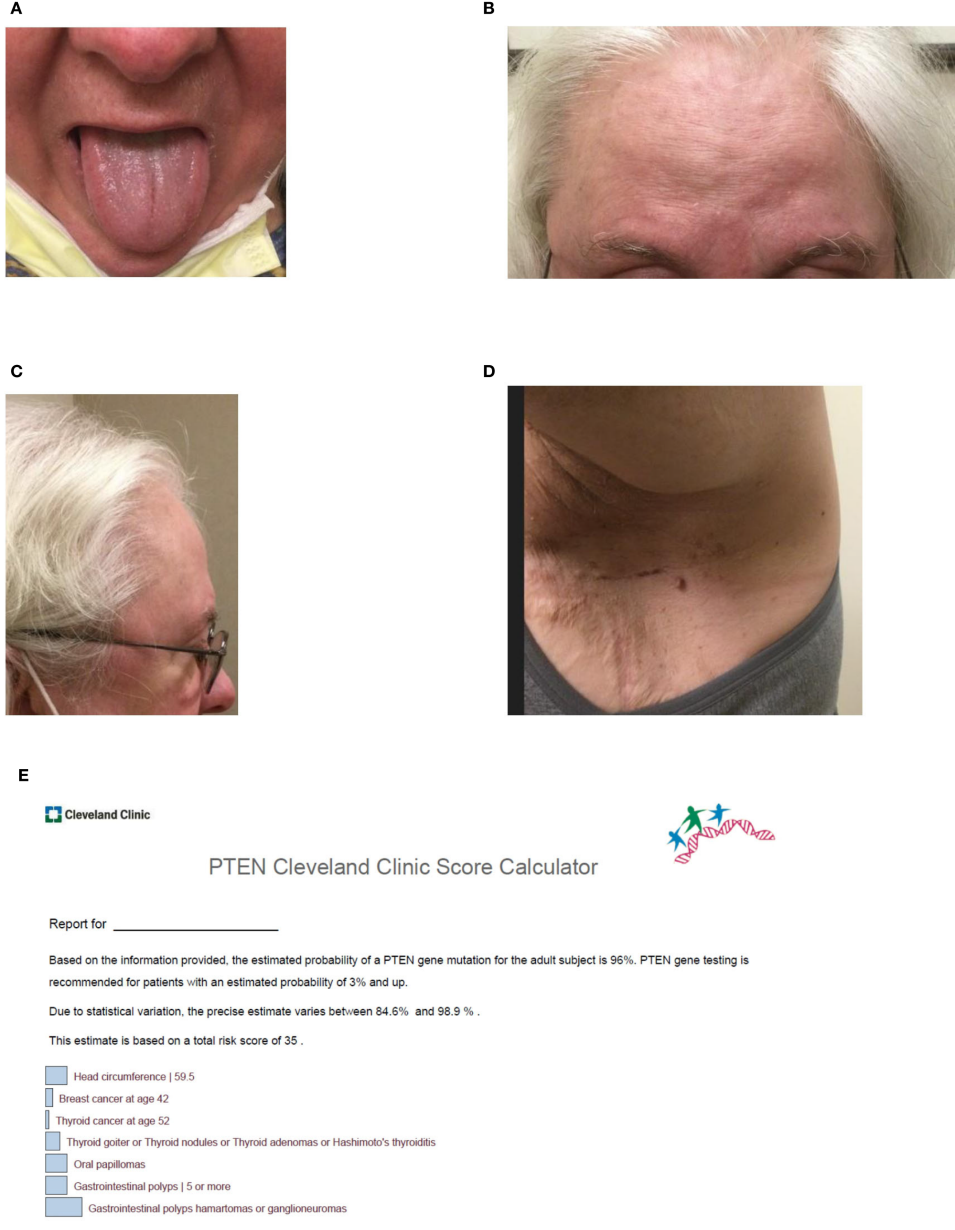
**(A–D)** Clinical features of Cowden syndrome including **(A)** lingual papules, **(B)** multiple trichilemmomas on the forehead, **(C)** frontal bossing contributing to macrocephaly with an occipital-frontal circumference of 59.5 cm, and **(D)** acrochordons of the axilla. At the time of the visit, a Cleveland Clinic PTEN risk score (6) was calculated as 35 for the patient (see **Figure 2E**). **(E)** Visual of the patient’s Cleveland Clinic PTEN risk score (6). The risk score which was calculated as 35 represents a 96% (84.6%-98.9%) predicted probability of identifying a pathogenic variant in *PTEN*.

On physical exam, the patient’s head circumference was 59.5cm, surpassing the threshold for macrocephaly in women (5). Tongue papules and trichilemmomas of the face were also present, as was frontal bossing (see **Figures 2A-D**). Acrochordons were also seen, especially in the axillary region. At the time of the visit, a Cleveland Clinic PTEN risk score (6) was calculated as 35 for the patient (see **Figure 2E**).

### Genetic testing

The patient met clinical criteria for PHTS/Cowden syndrome and was counseled on the possibility that germline testing would identify a mutation in the *PTEN* gene. Due to her family history of cancer and desire to have complete information after testing, she elected to proceed with testing with a 77-gene DNA multigene panel. The patient was tested at Ambry Genetics, Aliso Viejo, CA, USA, using CAP/CLIA-certified clinical bait-capture methodology. Capture-based RNA testing, run concurrently, was ordered to increase chances of finding a causal germline alteration after the potential for non-detection of some disease-causing mutations by DNA was explained to her, in particular that ~15% of classic CS and ~95% of CS-like individuals lack pathogenic *PTEN* mutations (7). Additional specifications are detailed in the clinical report (see **Supplementary Figure 1**).

Germline testing of the patient’s DNA showed a negative result. For RNA testing, RNA was isolated from the patient’s specimen using standardized methodology and quantified (8, 9). In this process, RNA is converted to complementary DNA (cDNA) by reverse transcriptase polymerase chain reaction (RT-PCR). Sequence enrichment of the targeted coding exons and adjacent intronic nucleotides is carried out by a bait-capture methodology using long biotinylated oligonucleotide probes followed by polymerase chain reaction (PCR) and next-generation sequencing. Any transcripts found are compared with a human reference pool. The absence or presence of RNA transcripts meeting quality thresholds is incorporated as evidence toward assessment and classification of DNA variants. Any regions not meeting RNA quality thresholds are excluded from analysis. In this approach, RNA sequencing was not used to identify the variant nor was it used to confirm it; it was used to identify the aberrant RNA splice defect (r.209_210ins209 + 1991_209 + 2042). Following this, targeted Sanger sequencing was performed in this region to identify the c.209 + 2047A>G variant. Once identified, RNA data were used to inform the clinical interpretation.

**Figure 3:**
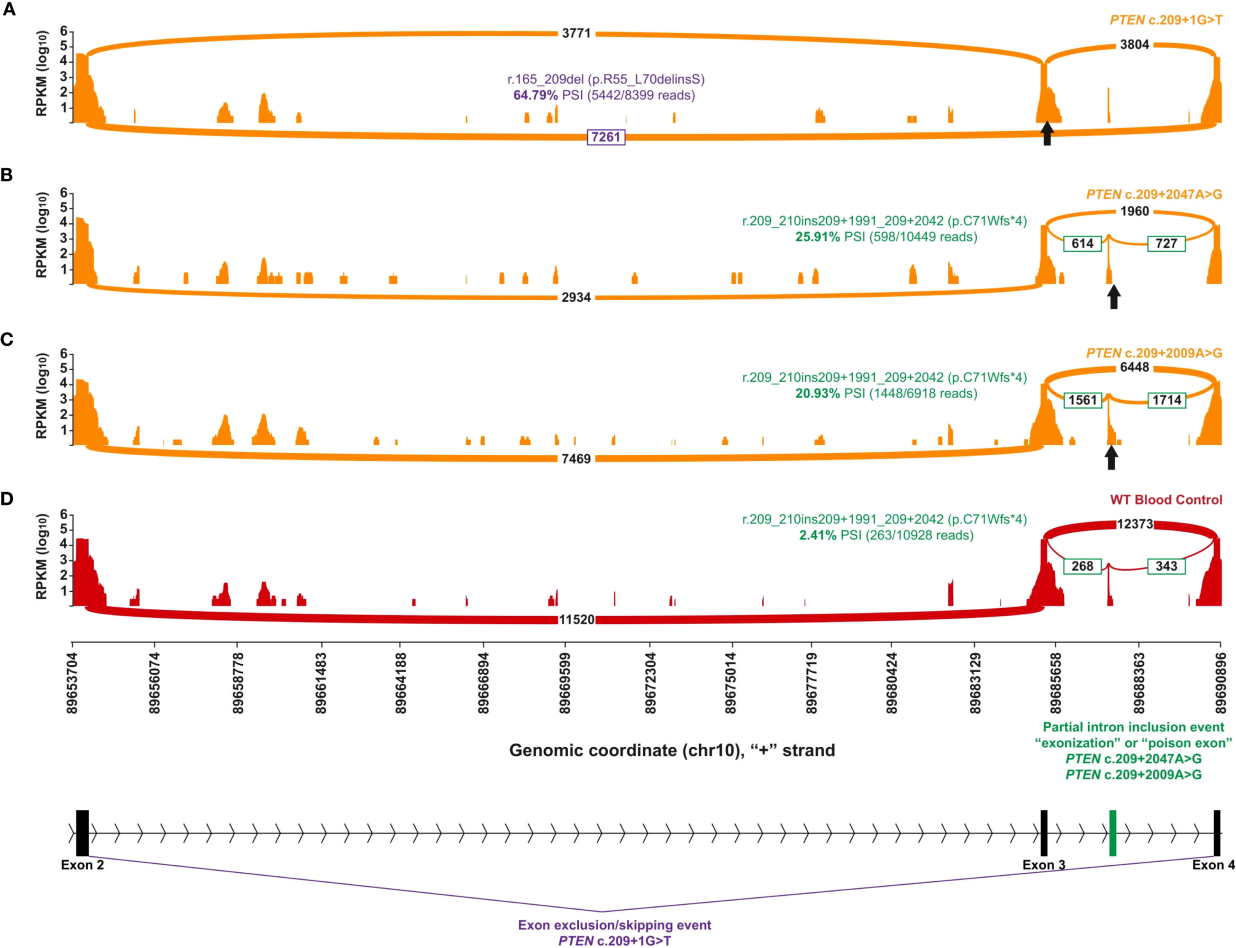
Capture RNA-Seq results of individuals with *PTEN* variants in intron 3. The orange plots represent individuals with different spliceogenic *PTEN* variants, whereas the red plot indicates a representative control that has no alterations in *PTEN*. The position of the different genetic variants is indicated by a black up-arrow. The identity of the most prominent aberrant transcript and the quantification are color-coded by aberrant splice effect. A scaled diagram of the exon–intron structure as well as the genomic coordinates (GRCh37) and summary of different splice events are indicated at the bottom with color coding to match parts **(A-D)**, where purple indicates exon 3 skipping and green indicates partial exonization of intron 3. **(A)** A positive control sashimi plot of an individual with *PTEN* c.209 + 1G>T, a canonical splice alteration at the donor site of exon 3 that causes skipping of exon 3 (in purple). **(B, C)** Sashimi plots of the individual with *PTEN* c.209 + 2047A>G **(B)** and an individual with a close match *PTEN* c.209 + 2009A>G **(C)**, both causing the same splice defect leading to partial exonization of intron 3 (in green). **(D)** A selected representative control expressing low levels of the same aberrant transcript identified in **(B, C)**.

RNA testing identified an A to G substitution 2,047 nucleotides into the intron following exon 3 (NM_000314.4:c.209 + 2047A>G), representing a deep intronic variation leading to alternative splicing of the *PTEN* RNA and the *PTEN* protein product (NP_000305.3). “Deep intronic” here is defined as beyond 30 nucleotides into the intron, which is the extent of the analytical range of intronic sequencing at the reference laboratory. The aberrant event in question created an exonization event, sometimes called a “poison exon,” where part of the intron was included in the open reading frame. This 52-base-pair-included intronic portion shifted the native reading frame of the transcript and led to a premature termination codon (NP_000305.3:p.C71Wfs*4) that is expected to undergo non-sense-mediated mRNA decay. The patient’s genetic testing DNA and RNA result (**Supplementary Figure 1**) and sashimi plot (**Figure 3**) showing the relative transcript quantity and location of the alternatively spliced RNA molecules associated with the patient’s *PTEN* intronic variation are shown.

## Discussion

*PTEN* is a tumor-suppressor gene located on chromosome 10q23 (10). PVs have been reported in all nine exons of *PTEN* with various types of PV, including missense, non-sense, and splice sites, as well as intragenic deletions or insertions and large deletions. In particular, PV in exons 5, 7, and 8 are overrepresented with three common non-sense PVs seen (p.R130*, p.R233*, and p.R335*). Intronic and deep intronic *PTEN* PVs have also been previously reported in the literature (10, 11). Prior analysis of 34 germline intronic variants of 61 patients and family members with suspected PHTS with mRNA analysis showed splice aberrations in 33 samples (12). This study was limited to intronic variants at positions −5 and +5 (five nucleotides upstream or downstream of an exon). Here, 15 intronic variants resulted in exon skipping. Other types of aberrant splice changes were also seen, including new/cryptic splice sites, splicing using cryptic splice sites within exons, and retention of intronic sequences. Notably, this analysis went a step further by also performing protein expression of the 33 germline *PTEN* intronic variants studied. However, it should be noted that there was no specific genotype–phenotype relationship identified (3). In addition, there is a broad estimate for the frequency of *de novo PTEN* mutations, with an estimate of anywhere from 10.7% to 47.6% (13).

Our patient presented for genetic risk assessment with several classic phenotypic features concerning CS/PHTS, including a history of bilateral breast cancer, thyroid cancer, colonic ganglioneuromatosis, as well as classic physical exam features for CS such as frontal bossing, macrocephaly, tongue papules, facial trichilemmomas, and axillary acrochordons. Our patient met testing criteria for CS/PHTS, with the fulfillment of three or more major criteria for CS (e.g., breast cancer, multiple GI ganglioneuromas, and macrocephaly as defined under the original consensus criteria) (14). Although mucocutaneous lesions were suspected, these were not biopsied, as ample criteria were already met to proceed with genetic testing.

The patient’s DNA testing result by panel testing was negative, but concurrent RNA testing identified an aberrant splice defect (*PTEN* r.209_210ins209 + 1991_209 + 2042) that led to the identification of the *PTEN* c.209 + 2047A>G deep intronic variant (**Figure 3**). The aberrant splice defect, r.209_210ins209 + 1991_209 + 2042, represents a 52-base-pair (bp) pseudoexon inclusion predicted to generate a premature termination codon (p.Cys71Trpfs*4) and a transcript subject to non-sense-mediated decay. This variant was classified as likely pathogenic based on the amount of aberrant splicing as well as the presentation of this and other patients (**Supplementary Figure 1**). A prior review of intronic *PTEN* alterations found that patients with this seemingly rare subtype of PV manifested more severe syndromic phenotypes reflected through higher Cleveland Clinic PTEN risk scores compared with those without detectable changes in RNA (12). Notably, the Cleveland Clinic PTEN risk calculator was developed to evaluate the pretest probability of harboring a germline *PTEN* alteration (6), with our patient having a score of 35 leading to an estimated 84.6%-98.9% risk. However, these findings point to a potential missed opportunity to identify intronic mutations in patients with clinical CS/PHTS after negative DNA testing, which could have substantial benefits in informing the risk stratification and prevention management of close relatives.

Significant opportunity exists to not only improve the yield of germline genetic testing in suspected PHTS but also reclassify variants of uncertain significance. In one study, RNA analysis assisted in the reclassification of 20% of splicing variants from VUS to PV (15). In another larger study, 43,524 individuals underwent paired DNA and RNA testing, leading to the detection of PV, including deep intronic alterations, resulting in identifying 87 individuals with a clinically actionable result (16). Furthermore, a recent large study of splicing variants in a 689,321-person clinical cohort demonstrated that among all individuals tested, 5.4% had splicing VUS and RNA analysis would be estimated to reclassify 1.7% of the individuals in the cohort (17). Paired DNA/RNA testing may also have more impact in underrepresented populations, with one study demonstrating medically significant reclassifications made in 1.3% of non-white and Hispanic individuals compared with 0.7% of non-Hispanic white (p<0.001). This increased the positive rate by 3.1% and decreased the VUS rate by 3.9% in non-white and Hispanic individuals (8, 9). Finally, RNA analysis may be useful beyond splicing and help further classify some truncating sequence variants and intragenic copy number variants and structural rearrangements (17). DNA sequencing panels that cover larger or entire intron regions could improve detection of deep intronic pathogenic variants. The introduction of long-read DNA sequencing and clinic whole-genome sequencing (WGS) by commercial laboratories also have the potential to improve detection of deep intronic pathogenic splice variants. However, the challenge of interpreting variants identified in intronic regions still exists in the absence of RNA data, regardless of the completeness of sequencing coverage. In the future, WGS coupled with whole transcriptome sequencing could improve efficiency and accuracy of disease-causing alterations.

Important limitations of this report include the greater ease of detection of exon skipping events relative to inclusion events as well as the diagnostic blind spot around quantifiable large intron retention events. While the generalizability of the prevalence of this rare alteration may be questioned, query of the gnomAD database, which includes both genomes and exomes with good coverage in the *PTEN* region in question, reports no findings among nearly 152,000 alleles sequenced. Finally, while RNA expression variability can be a limitation in RNA-based clinical testing, RNA expression for *PTEN* is high in the blood, leading to high-quality data and high confidence from the diagnostic laboratory standpoint of variant detection using RNA CaptureSeq technology.

Concurrent to our patient’s evaluation and publication of this case report, RNA analysis performed by another research team identified the same deep intronic variant in *PTEN*, in a 59-year-old male patient with no family history of cancer presenting with multiple juvenile polyps on colonoscopy (NM_000314.4:c.209 + 2047A>G). Notably, the patient had a history of macrocephaly, thyroid cyst/goiter, and upper GI polyps but lacked a history of thyroid cancer or skin abnormalities. The RNA analysis performed on this patient revealed that the identified PV led to a 52-bp pseudoexon inclusion by impacting a cryptic splice donor site (18). This patient was recommended to pursue screening based on Danish PHTS guidelines, which recommends colonoscopy every 5 years but does not recommend any upper GI screening (19). RNA analysis in this case also led to a significant change in clinical recommendations and classification of this finding as a PV. One of the important implications of our case and this one is that RNA-based identification of a PV allows family members to gain the ability to quantify their cancer risk. More recently, a case report has identified another 25 year-old patient with clinical features consistent with PHTS/Lhermitte–Duclos syndrome, including thyroid abnormalities, extremity angiolipoma and xanthomas, breast fibroadenomas, lingual papillomatosis, neurological symptoms, and macrocephaly, among other findings (20). Cleveland Clinic score (6) was 28 with a 77% chance of having a germline *PTEN* mutation, but testing by a 13-gene hereditary breast ovarian cancer panel showed a negative result. Further genetic testing using whole-genome sequencing of the patient and two family members and targeted RNA analysis identified a deep intronic variant, c.492_1671_492 + 1768del, leading to a 98-bp deletion, as the underlying cause of the patient’s PHTS.

In conclusion, many families with phenotypic PHTS/Cowden syndrome have a negative germline DNA testing result, but this result may miss deep intronic variants that affect RNA splicing and protein expression and/or function. Our case highlights the improved yield with an approach of concurrent DNA/RNA testing; however, the absolute number of patients with clinical PHTS that could be molecularly diagnosed by RNA remains unknown. Nonetheless, the relevance of further studies to determine the prevalence of deep intronic variants in individuals/families meeting clinical PHTS criteria is critical, as the testing may have downstream implications for cancer prevention and early detection for multiple family members across future generations. Our case illustrates the critical need to consider restesting patients meeting PHTS criteria but negative by DNA testing with additional novel diagnostics like RNA testing to maximize detection of genetic variants that drive hereditary cancer risk. As paired DNA/RNA analysis is now routinely available through multiple germline genetic testing laboratories, we suggest ordering paired DNA/RNA testing upfront on patients who are phenotypically suspicious for PHTS to avoid missing a potential deep intronic PV. In addition, our case illustrates the critical need to consider retesting patients meeting PHTS criteria who are negative by DNA testing with additional novel diagnostics like RNA testing to maximize detection of genetic variants that drive hereditary cancer risk.

## Data Availability

The datasets presented in this study can be found in online repositories. The names of the repository/repositories and accession number(s) can be found in the article/**Supplementary Material**.
