# Supplementary material for: Paired DNA/RNA testing uncovers a deep intronic PTEN pathogenic variant associated with clinical Cowden Syndrome: a case report
Source: Front Oncol. 2025 Oct 9;15:1679432. doi: 10.3389/fonc.2025.1679432 (PMC12545062; doi:10.3389/fonc.2025.1679432)
Supplement: Supplementary file 2 [file DataSheet2.pdf]

FIGURE 3a. Patient's DNA and RNA genetic testing result

BRCA1/2 Analyses with CancerNext-Expanded® +RNAinsight®

RESULTS	
PTEN	**Variant, Likely Pathogenic: c.209+2047A>G**

SUMMARY

POSITIVE: Likely Pathogenic Variant Detected (See RNA Impact below)

INTERPRETATION

- This individual is heterozygous for the **c.209+2047A>G** likely pathogenic variant in the *PTEN* gene.
- This result is consistent with a diagnosis of *PTEN* hamartoma tumor syndrome (PHTS).
- **Risk estimate**: lifetime risks of up to 85% for breast cancer (females only), up to 38% for follicular thyroid cancer in females (6% in males), up to 28% for endometrial cancer (females only), up to 34% for renal cancer, and up to 20% for colorectal cancer.
- RNA Impact:
  - **Familial testing: this *PTEN* alteration is specifically identified through RNA testing, and the lab should be contacted to ensure accurate testing for family members.**
- The expression and severity of disease for this individual cannot be predicted.
- Genetic testing for likely pathogenic variants (VLPs) in family members can be helpful in identifying at-risk individuals.
- Genetic counseling is a recommended option for all individuals undergoing genetic testing.

No additional pathogenic mutations, variants of unknown significance, or gross deletions or duplications were detected. Genes Analyzed (77 total): ***AIP, ALK, APC, ATM, BAP1, BARD1, BLM, BMPR1A, BRCA1, BRCA2, BRIP1, CDC73, CDH1, CDK4, CDKN1B, CDKN2A, CHEK2, DICER1, FANCC, FH, FLCN, GALNT12, KIF1B, LZTR1, MAX, MEN1, MET, MLH1, MSH2, MSH6, MUTYH, NBN, NF1, NF2, NTHL1, PALB2, PHOX2B, PMS2, POT1, PRKAR1A, PTCH1, PTEN, RAD51C, RAD51D, RB1, RECQL, RET, SDHA, SDHAF2, SDHB, SDHC, SDHD, SMAD4, SMARCA4, SMARCB1, SMARCE1, STK11, SUFU, TMEM127, TP53, TSC1, TSC2, VHL* and *XRCC2*** **(sequencing and deletion/duplication);** ***AXIN2, CTNNA1, EGFR, EGLN1, HOXB13, KIT, MITF, MSH3, PDGFRA, POLD1*** **and** ***POLE*** **(sequencing only);** ***EPCAM*** **and** ***GREM1*** **(deletion/duplication only)**. RNA data is routinely analyzed for use in variant interpretation for all genes.

***PTEN* Additional Information**

The **c.209+2047A>G** intronic variant results from an A to G substitution 2047 nucleotides after coding exon 3 in the *PTEN* gene. This alteration has been observed in at least one individual with a personal and/or family history that is consistent with PTEN hamartoma tumor syndrome (Ambry internal data). This nucleotide position is well conserved on limited sequence alignment. *In silico* splice site analysis predicts that this alteration will result in the creation or strengthening of a novel splice donor site. RNA studies have demonstrated that this alteration results in abnormal splicing in the set of samples tested (Ambry internal data). Based on the majority of available evidence to date, this variant is likely to be pathogenic.
